# Being friendly: paced mating for the study of physiological, behavioral, and neuroplastic changes induced by sexual behavior in females

**DOI:** 10.3389/fnbeh.2023.1184897

**Published:** 2023-09-28

**Authors:** Elisa Ventura-Aquino, Raúl G. Paredes

**Affiliations:** ^1^Escuela Nacional de Estudios Superiores, Unidad Juriquilla, Universidad Nacional Autónoma de México, Querétaro, Mexico; ^2^Instituto de Neurobiología, Universidad Nacional Autónoma de México, Querétaro, Mexico

**Keywords:** paced mating, positive affective state (reward), motivation, opioids, rats

## Abstract

Paced mating in rats is an experimental condition that allows the evaluation of sexual behavior in a way that closely resembles what occurs in seminatural and natural conditions enabling the female to control the rate of the sexual interaction. In conventional non-paced mating tests, females cannot escape from male approaches, which may lead to an unrewarding overstimulation. Paced mating is an alternative laboratory procedure that improves animal welfare and has a higher ethological relevance. The use of this procedure contributed to the identification of physiological and behavioral factors that favor reproduction. Paced mating includes motivational and behavioral components differentiating quantitative and qualitative characteristics that are critical for the induction of the rewarding properties of mating. These positive consequences ensure that the behavior will be repeated, favoring the species’ survival. Sexual reward is an immediate consequence of paced mating, mediated mainly by the endogenous opioid system. Paced mating also induces long-lasting neuroplastic changes, including gene expression, synthesis of proteins, and neurogenesis in sex-relevant brain areas. The interest in paced mating is growing since the complexity of its elements and consequences at different levels in a laboratory setting resembles what occurs in natural conditions. In this review, we analyze the classic studies and recent publications demonstrating the advantages of using paced mating to evaluate different aspects of sexual behavior in females.

## 1. Introduction

**Figure 1:**
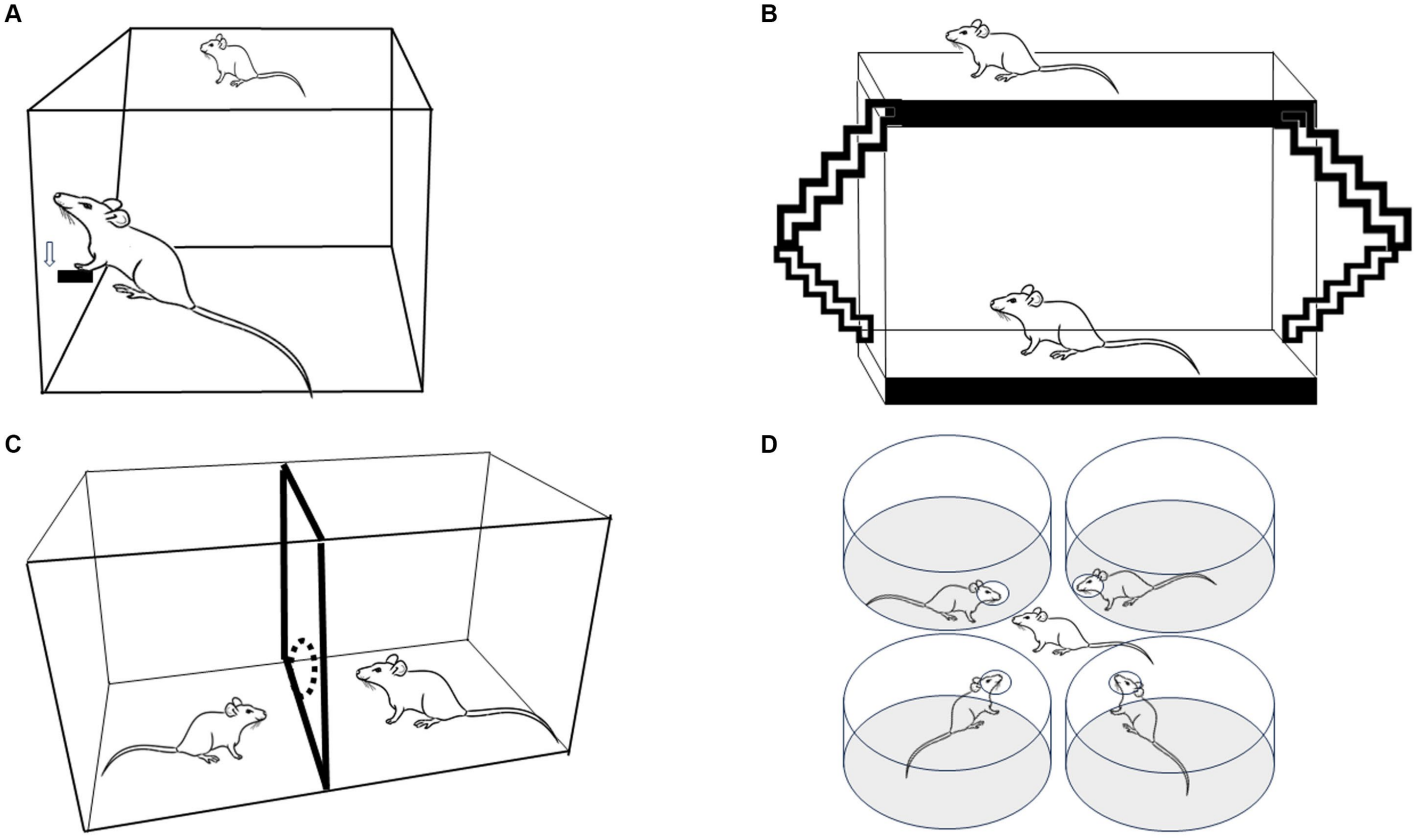
Four experimental strategies used to evaluate paced mating in female rats. **(A)** Lever press to obtain the presence of a sexually active male. **(B)** The bilevel chamber in which the females use the ramps to move up and down, changing the level in the arena. **(C)** The two-compartment arena in which females can pace the sexual interaction crossing through a hole that communicates both compartments. **(D)** Multiple partner preference choice.

Scientific studies about sexual behavior started in the late 18th century, with outstanding growth in the post-Second World War period. These early rodent studies mainly focused on behavioral elements and the rewarding properties of mating in males (Agmo, 2007a). At that time, mating tests were usually conducted in a standard cage where the male controlled the rate of sexual interactions (non-pacing, NP). When tested under that condition, female sexual behavior remained relatively stable until they showed rejection and avoidance behaviors, questioning whether sexual interaction was rewarding for females. Early experimental reports employing operant tasks demonstrated that sexually receptive females showed high motivation to access a male. Females were trained to lever press to have access to a male with whom to mate (Figure 1A). The return latencies to the lever were inversely related to the amount of stimulation the females received. In particular, latencies were shorter after a mount alone than after an intromission and both post-mount latencies and post-intromission latencies were shorter than after an ejaculation, which generally takes place after several intromissions (Bermant, 1961; Bermant and Westbrook, 1966). The authors concluded that females could work on spacing the stimulation they received, making the sexual contacts positively reinforcing (Bermant and Westbrook, 1966). Reinforcement refers to the increase in the probability that a response will be repeated, while reward refers to the ability to elicit an approach behavior to an incentive. In the case of mating, the incentive a male or female induces approach behavior in the appropriate hormonal conditions see Paredes (2009, 2014) for a discussion.

Other studies failed to demonstrate that sexual behavior could be reinforcing for female rats. For example, when female rats were trained in a straight runway to interact with different stimulus animals, females in estrous ran faster than anestrous females to interact with sexually active males. However, the estrous females ran equally fast to interact with sexually active or passive males. The interpretation of these results was that mating was not reinforcing for female rats and that social interaction was the main reward (Bolles et al., 1968). There were clear differences in the methods used in the two studies. With the lever press, the females can indeed pace the sexual interaction depending on the type of stimulation they receive. In the alley running, females received sexual stimulation every 10 min. Therefore, they were not able to pace the sexual interaction. Moreover, since they could run to a sexually or passive male, social contact could confound the interpretation of the results. Aside from operant tasks, other methods such as crossing an electrified grid, partner preference, and conditioned place preference (CPP) have clearly demonstrated that sexual interactions under appropriate conditions are appetitive to female rats see Paredes and Vazquez (1999) for a review.

It was not until the development of a standardized method to allow the female to control the sexual interaction (paced mating; PM) that this type of question could be addressed systematically. One of the key advantages of PM, developed by Mary Erskine (1946–2007) in the 1980s (Erskine, 1985, 1987, 1989), is that it partially resembles what occurs in the wild. This methodology allows researchers to evaluate different aspects of female sexual behavior under laboratory conditions. Since the 1980s, the number of studies employing PM has significantly grown, making this method a valuable tool to increase our understanding of the motivational and rewarding properties of mating in females. The consequences of PM at the reproductive and neuroplastic levels have also been explored, undoubtedly opening a new field of study on sexual behavior in females, a topic almost ignored previously.

In the following sections, we will describe the behavioral elements of female sexual behavior in the rat to continue with a general description of PM. We will then briefly review studies demonstrating that PM induces a reward state mediated by opioids. We will also describe the long-term plastic changes in neurogenesis induced by PM. We will explore how PM is employed to evaluate pharmacological strategies and their consequences on female sexual behavior. Finally, we will briefly mention how PM can be combined with magnetic resonance imaging to study the neural circuits controlling sexual behavior. Most of the reports were conducted in ovariectomized (ovx) and hormonal-primed rats. Otherwise, corresponding specifications will be noted.

## 2. Sexual behavior in the female rat

Observations in the laboratory, as well as in seminatural conditions, of female sexual behavior in rats identified that they display periodic solicitations, also called proceptive, appetitive, or paracopulatory behaviors, that influence the rate of mating by triggering mounts from the male (Erskine, 1989). Proceptive behaviors in the female rat include ear wiggling, hopping, darting around the male, and approaching and withdrawing movements. Proceptive behaviors have been considered as a motivational index and fluctuate along mating tests, i.e., they decay as sexual stimulation is extended but increase with the presentation of a novel sexual partner (Ventura-Aquino and Fernández-Guasti, 2013). The consummatory element, the lordosis posture, consists of the spinal dorsiflexion and elevation of the rump when the male mounts the female. This position enables the male to insert the penis into the female’s vagina (vaginal intromission) and reflects the level of sexual receptivity of the female. Usually, receptivity is measured by calculating the lordosis quotient (LQ), which is the percentage of mounts that trigger the lordosis posture in the female. Additionally, there is a four-level scale of lordosis rating (0: absent to 3: exaggerated) depending on its degree (lordosis intensity, LI) (Hardy and DeBold, 1972). Usually, lordosis indicates the sexual responsiveness of the female, and it stays stable as long as the receptivity period lasts (Agmo, 2007b; Blaustein, 2009; Ventura-Aquino and Fernández-Guasti, 2013).

## 3. PM components

PM is a method very easy to set up since it does not require sophisticated equipment; it is inexpensive, and it is very reliable. The protocol requires only an observation cage (which can be made up of Plexiglass), females in the appropriate hormonal condition and a sexually trained male to pace the sexual interaction. Paced mating has been tested in cycling females in proestrus and Ovx hormone-primed subjects showing similar behavioral patterns with some differences depending on the hormonal scheme (Brandling-Bennett et al., 1999; Zipse et al., 2000). Most PM studies are conducted in an arena made of clear Plexiglass (40 × 60 × 40 cm), divided in two by a removable partition with one hole at the bottom, 4–7 cm in diameter, as described initially by Erskine (1985, 1989) and Figure 1C. In this way, the female can go back and forth between both compartments, whereas the male, which is usually bigger, cannot go through the hole when pursuing the female. When the male is about the same size as the female, he can be trained to stay in his compartment by gently tapping him on the nose (Erskine, 1985, 1989). Some studies have used a partition with two, three, or four holes to allow the female to move from one compartment to the other (Erskine and Hanrahan, 1997; Rossler et al., 2006; Coria-Avila et al., 2008; Snoeren et al., 2011). It has been reported that females who pace in a four hole mating cage show a shorter interintromission interval (Yang and Clemens, 1996) and a higher number of hops and darts (Ismail et al., 2008, 2009). In our experience (Camacho et al., 2009a), the sexual behavior parameters are the same if the females pace the sexual interaction through one or three holes. Moreover, both with one hole or three hole partitions, a positive affective reward state is induced, suggesting that pacing the sexual interaction through one or more holes has the same consequences on female sexual behavior as revealed by a subsequent conditioned preference for the mating chamber (Camacho et al., 2009a). Males appear to be more sensitive to the context in which copulation occurs, showing more behavioral differences if they mate in 1 or 4 hole pacing chambers (Ismail et al., 2008, 2009).

Different parameters can be obtained from paced mating tests. For instance, it is possible to calculate the percentage of exits (%E) after receiving mounts (%EM), intromissions (%EI), or ejaculations (%EE), but also the time that the female takes to return to the male side, defined as return latencies after mounts (MRL), intromissions (IRL) or ejaculations (ERL). The %E represents the female’s ability to discern the stimulation received and correlates with the stimulation intensity, i.e., %EM is lower than %EI, and both %EM and %EI are lower than %EE.” On the other hand, latencies to return are considered indicators of the female motivation to resume mating, i.e., shorter latencies reflect higher motivation and vice versa (Erskine, 1992; Coopersmith et al., 1996).

In traditional mating tests, rejection and aggressive behaviors towards the male can be observed when testing is extended and the females have received repeated stimulation or when the estrous period is finishing, reflecting a lowering of sexual motivation in females. Prolonged vaginal penetration increases the frequency of rejection behaviors and reduces the intensity and probability of subsequent lordosis (Bermant and Westbrook, 1966; Hardy and DeBold, 1972). Moreover, lordosis is inhibited after a brief period of intensive mounting by the male rat (Hardy and DeBold, 1972). These studies indicate that sexual interaction in the female rat has appetitive and aversive components. The aversive properties of mating are highly reduced when females pace their sexual contacts (Erskine, 1989; Paredes and Alonso, 1997; Paredes, 2009).

### 3.1. Other methods in which females control the rate of sexual stimulation

The original method described by Erskine, allowing the female to control the sexual stimulation received, significantly contributed to the understanding of the behavioral and physiological advantages of paced mating. Other groups modified the method, always allowing the female to control the sexual stimulation, to analyze different components of female sexual motivation. The modified methods are the bilevel chambers and the multiple partner preference/choice test.

In the bilevel chambers (51 × 70 × 15 cm boxes made of Plexiglas), the female can pace the sexual interaction by forcing the males to chase them while they run between levels, as shown in Figure 1B, Mendelson and Gorzalka (1987), Pfaus et al. (2000), and Pfaus et al. (1999). Moreover, animals can use ramps to move from one level to the other. The narrow chamber keeps the animals in a side-ways position, which is optimal for viewers. Measures registered in this method are anticipatory level changing before the introduction of the male (considered as sexual motivation parameter), latency and frequency of proceptive behaviors, lordosis quotient, lordosis intensity, and the number of rejections (Pfaus et al., 1999).

The multiple partner preference/choice test is another model in which females control the stimulation they receive during sexual interaction. The arena comprises four cylinders, each with a hole facing the central zone. Each cylinder contains a tethered sexually experienced male stimulus animal that can display sexual behavior but cannot leave the cylinder (Figure 1D). In this way, the female can choose to interact with any of the four males (Ferreira-Nuno et al., 2005). As occurs in paced mating tests, the percentage of exits after intromission or ejaculations is higher than the percentage of exits after mounts. The females spent a significantly longer time with a preferred male (Ferreira-Nuno et al., 2005), demonstrating again that females can discriminate and select sexual stimulation.

It should be clear by now that the ability of females to control or pace the sexual interaction can be observed using different methodologies including bar pressing (Bermant, 1961; Peirce and Nuttall, 1961; Bermant and Westbrook, 1966), mating with tethered males (Edwards and Pfeifle, 1983), bilevel chambers (Mendelson and Gorzalka, 1987; Pfaus et al., 1999, 2000), multiple partner preference/choice arena (Ferreira-Nuno et al., 2005) and paced mating with one hole (Erskine, 1989; Paredes and Alonso, 1997) or four holes (Yang and Clemens, 1996; Yang and Clements, 2000). Fewer rejections behaviors are observed using these methodologies, indicating reduced aversive stimulation. One important characteristic of these different methods used in laboratory conditions is that the females display behavioral patterns similar to those observed in seminatural or natural conditions (Barnett, 1975; Mcclintock and Adler, 1978; Mcclintock and Anisko, 1982), including solicitations, hopping and darting. The possibility to study the sexual interactions in natural or seminatural environments allows a more natural context and a fine-tuned analysis of this motivated behavior but PM remains the best option when rodents are tested in a laboratory condition.

Early classical studies demonstrated that the copulatory pattern of male and female rats in the wild and seminatural conditions is promiscuous. Estrous is synchronized among females, and mating occurs in groups with several males and females (Barnett, 1975; Robitaille and Bovet, 1976; Mcclintock and Adler, 1978; Mcclintock and Anisko, 1982). In group mating, males and females repeatedly change partners. For the female, there is no order sequence of stimulation they received. Several intromissions do not necessarily precede an ejaculation. A female can start mating with a male who has intromitted several times with other females and can receive an ejaculation without previous intromissions. In group mating, males and females experience the same amount of copulation (Mcclintock et al., 1982). Recent studies by Chu and Agmo (2015), Chu et al. (2017), and Hernandez-Arteaga and Agmo (2023) have evaluated sexual behavior in seminatural conditions. The arena consists of an open area with tunnels and burrows where 3 males and 4 female rats are housed together for several days. When tested under these conditions, females prefer a specific male, receiving more intromissions and ejaculations from this male than from other males (Chu and Agmo, 2015; Chu et al., 2017). It is clear that in group mating in seminatural or natural conditions, both sexes control the rate of sexual interaction, receiving a sufficient amount of stimulation from one or several members of the opposite sex, which makes sex rewarding and sexual behavior repeated in the future see Martinez and Paredes (2001) and Paredes (2009, 2014) for a discussion.

### 3.2. PM in mice

Although most pacing studies have been performed in rats, few studies have evaluated PM in mice following a similar methodology. Since size differences between male and female mice are not as evident as that observed in rats, in one study, the authors used a Plexiglass barrier (10 cm tall) to divide the male from the female side. The male was tethered and could not leave his side of the cage, while the female mice could jump the barrier to be with the male. Female sexual behavior was compared when they mated in the PM and NP paradigms. The authors found that, like female rats, female mice can pace the sexual interaction. They took longer to return to the male side after an ejaculation than after a mount or an intromission (Johansen et al., 2008).

In another study, the authors used a similar design as that used in rats with a Plexiglass partition with four holes at the bottom of sufficient size to allow the female mice but not the male to move from one side to the other. The authors used significantly smaller females, around 25 g, than males, around 45 g (Farmer et al., 2014). One important point that needs to be considered when evaluating PM in female mice is that proceptive behaviors are not so evident as in rats. Acceptances, defined as the percentage of approaches by the male that terminate in mounts or intromissions, are used as a measure of sexual receptivity in female mice (Johansen et al., 2008). Unfortunately, the behavioral, physiological, and neuroplastic changes induced by PM in mice have not been studied as much as in rats. This clearly represents an opportunity for future studies.

Another important contribution of the paced mating method is that it allows a clear dissociation of the appetitive components of female sexual behavior, leading to an understanding of the rewarding aspects of this behavior. Moreover, what we have learned about paced mating and sexual reward has also contributed to our general understanding of reward and conditioning. In the following section, we will describe the rewarding aspects of paced mating.

## 4. PM, reward, and neuroplasticity

### 4.1. PM and reward

A relevant contribution of PM studies is the demonstration of a reward state after mating in females, evaluated by the conditioned place preference (CPP) paradigm (Paredes and Alonso, 1997; Camacho et al., 2009a). CPP is conducted in an arena divided into three compartments, two with distinctive and contrasting characteristics and a neutral one in the middle. The CPP evaluates approach behavior towards environments associated with a previous reinforcing event (food, drug, sex). In the pre-test, the preferred compartment is determined on the basis of the times spent in each of the two lateral compartments. Later, the animal is placed in the preferred compartment without any reinforcing stimulus. On alternate days, the female mates and immediately thereafter, is placed in the non-preferred (reinforced) compartment. In this way, the state induced by mating is associated with the non-preferred compartment. After three non-reinforced and three reinforced sessions, the preference is tested again. The change of the original preference is widely accepted as an objective way to determine the induction of a positive conditioned affective reward state. In the case of mating, the preference change by PM is similar to that induced by a dose of morphine (1 mg/kg) in both sexes (Camacho et al., 2009b; Arzate et al., 2011). Robust evidence indicates that PM is rewarding in both sexes. That is, males and females, need to control the rate of sexual interactions to find sex rewarding. More specifically, females need to mate in a pacing chamber, and males need to control the access to the female (Martinez and Paredes, 2001). However, some studies have described sexual reward in NP conditions. For example, Oldenburger et al. (1992) evaluated CPP in females after six conditioning sessions where mating occurred in the non-preferred compartment alternated with sessions where females stayed alone in the other compartment in counterbalance sessions (Oldenburger et al., 1992). Only a weak effect on conditioning was observed. The authors compared the time spent in the compartments in 5-min epochs, finding statistical differences only in the last 5-min period (Oldenburger et al., 1992). When mating occurs in the conditioning cage, the females can associate both the appetitive and aversive components of mating, reducing the effect of conditioning.

A study by Meerts and Clark (2007) reported that females develop CPP in NP conditions. Two experiments were performed. In experiment 1, females could pace or not the sexual interaction until they received 15 intromissions, including ejaculations. Both groups developed CPP. In experiment 2, one group of females received 15 paced intromissions with the same male, and another group received the same number of intromissions with different males. In this case, females developed CPP only when a single male provided the stimulation, but not when the male was replaced after the first ejaculation with a second one (Meerts and Clark, 2007). In a follow up study, they found that artificial vaginal cervical stimulation (VCS) induces CPP (Meerts and Clark, 2009). In fact, the importance of timing the stimulation to induce sexual reward in females was demonstrated by Jenkins and Becker (2003a). They lengthened the sexual stimulation in NP by retiring the male after each intromission to mimic the interintromission interval observed in PM conditions (Jenkins and Becker, 2003a). Females developed CPP in PM conditions when the male was removed to mimic the female preferred interval (Jenkins and Becker, 2003a). One possible explanation for the different results between our studies and those by Meerts and Clark is that the males used in their studies ejaculated after around 6 intromissions, and our males required around 10–12 intromissions to ejaculate. On average, their females received about 2 ejaculations, because their females received 15 intromissions before they were placed in the conditioning cage (Meerts and Clark, 2007). The postejaculatory intervals, at least two for each female, could reduce the aversive components of NP, enhancing the rewarding effects. Another possibility is that Long-Evans rats (used in the studies by Meerts and Clark) are more sensitive to the appetitive effects of mating than the Wistar rats used in our studies. In fact, when female Wistar rats mate in PM conditions with the same male until receiving 15 intromissions, a clear CPP is observed. However, no CPP is produced if the female mates in NP conditions with the same male (Camacho et al., 2009a).

Another critical aspect of PM is the amount of stimulation required to induce the reward state. In females, at least 10 intromissions (with or without ejaculation) are needed (Paredes and Vazquez, 1999; Martinez and Paredes, 2001; Camacho et al., 2009b). When PM is extended to around 25 intromissions, CPP is still present independently of the number of ejaculations received (Arzate et al., 2011). For males, 15 intromissions or ejaculation are required to induce CPP.

### 4.2. Reward state induced by PM is mediated by opioids

The reward state induced by PM is prevented by the systemic administration of naloxone, an opioid receptor blocker, in males (16 mg/kg; Agmo and Berenfeld, 1990; Agmo and Gomez, 1993) and females (4 mg/kg; Paredes and Martinez, 2001). Similarly, when naloxone is infused directly into the medial preoptic area (MPOA), the ventromedial hypothalamus (VMH) and the amygdala (AMG) of females, CPP is also blocked suggesting a central role of opioids in sexual reward (Garcia-Horsman et al., 2008).

We also evaluated if sexual behavior could induce a reward state of the same intensity as a morphine injection. One group of females was allowed to pace the sexual interaction before being placed in the non-preferred compartment. In alternate sessions, they received a morphine injection before being placed in the preferred compartment. A second group received the reversed treatment. Only the females placed in the originally non-preferred compartment after paced mating developed CPP, suggesting that paced mating induces a reward state of higher intensity than a morphine injection of 1 mg/kg. In the same study, we also demonstrated that females that pace the sexual interaction for 1 h continue mating and develop CPP. No CPP was observed in the females that mated for 1 h without pacing the sexual interaction (Arzate et al., 2011), further demonstrating the biological relevance of the female’s ability to space the coital stimulation received during mating.

### 4.3. PM and neuroplasticity

We have also demonstrated that PM induces permanent neuroplastic changes in Ovx females hormonally primed with estradiol benzoate (EB) and progesterone (P). For example, a single PM session is enough to promote newborn cells in the granular layer of the accessory olfactory bulb (AOB), evaluated 15 days later. This effect was blocked by administering naloxone (4 mg/kg/i.p.), suggesting that opioids have an essential role in neurogenesis induced by PM (Santoyo-Zedillo et al., 2017). Subsequent studies showed that after four sessions of PM, one session per week, females showed more newborn cells integrated into the granular and the mitral layers of the AOB when they paced the sexual interaction compared to females that mated without pacing and to a control group. Moreover, after 10 PM sessions, one per week, the number of cells in the glomerular layer of the AOB and the granular layer of the MOB was higher compared to control and NP groups at day 45 (Alvarado-Martínez and Paredes, 2018; Portillo et al., 2020). These studies clearly indicate that PM induces long-term plastic changes that could explain this mating condition’s behavioral and physiological changes (Bedos et al., 2018). To date, the functional implications and relevance of PM induced neurogenesis are still a matter of study.

## 5. Sensory pathways important for PM

Whenever a behavior induces a reward state, it is more likely to be repeated in the future, and in the case of mating, this eventually impacts the species’ survival. In this regard, genitosensory stimulation under PM is qualitatively and quantitatively different from NP. For example, PM intromissions are usually longer (616 ± 30 vs. 527 ± 30 msec) than in NP conditions, suggesting that PM intromissions are a more intense stimulus than NP intromissions (Erskine et al., 1989). Additionally, steroids hormones, i.e., EB and P increase the responsiveness to stimulation by enlarging the field of the pudendal and pelvic nerves, which corresponds to the cutaneous areas of the flanks, perineum, clitoral sheet, and the caudal reproductive and urinary systems, including vagina, cervix, and bladder (Komisaruk et al., 1972). Thus, the female’s capability to discern the type and intensity of sexual stimulation received highly depends on the neural input. For example, a study evaluated PM in ovariectomized females 14 days after transection of pudendal (Pu), pelvic (Pe), or pudendal + pelvic (PuPe) nerves. Females were treated with EB for 7 days or with EB + P for 14 days. After treatment with EB, all groups (Pu, Pe, and PuPe) showed decreased pacing behavior compared to sham controls. When EB + P was administered, only the Pe and PuPe groups showed a reduction in pacing behavior (Erskine, 1992). These results suggest that P reduces the effects of autonomic nerve transection, increasing the threshold of the VCS favoring the return to the male side with a shorter latency. They also indicate that the afferent inputs from the vagina, cervix, and the surrounding skin via the pelvic and pudendal nerves to the spinal cord are relevant for the display of the pacing pattern in association with ovarian hormones, especially in combination with the well documented antinociceptive effect of P (Meyerson, 1967; Gilman and Hitt, 1978; Kim et al., 2012; Hornung et al., 2020). This combination of sensory stimulation and ovarian hormones allows the female to discern the stimulation they receive during mating and contributes to the physiological and behavioral consequences induced by PM.

## 6. Neuroendocrine responses induced by PM

### 6.1. Prolactin

The VCS received under PM is critical to trigger neuroendocrine responses. For example, in PM conditions, intromissions induce a twice-daily prolactin surge activating the luteal function and abbreviate the receptivity period favoring pregnancy. In NP mating conditions, more intromissions are required to generate the same physiological changes (Erskine and Kornberg, 1992). The prolactin release is correlated with the induction of pregnancy or pseudopregnancy independently of the mating condition. However, PM is more efficient in inducing this response because noradrenergic neurons convey genitosensory inputs to mating-responsive forebrain areas such as the medial amygdala that projects to the VMH where prolactin is released (Erskine and Hanrahan, 1997; Northrop et al., 2010). Additionally, the role of PM favoring reproduction is shown in intact females that mate under PM conditions which have bigger litters than females mated under NP conditions (Coopersmith and Erskine, 1994).

### 6.2. Progesterone and oxytocin

Mating under both conditions induces similar acute increases in progesterone (P) and 5 alpha-Androstane-3 alpha, 17 beta-diol (3 alpha-Diol), suggesting that PM and NP cause similar levels of stress (Frye et al., 1996). When PM was tested in combination with ovarian hormones, no effect on basal anxiety was found for sexual history, while an anxiolytic effect was found for progesterone (Arnold et al., 2019). Nevertheless, in the same study it was found that paced mating reduced anxiety after an acute stressor, suggesting that PM provides an increased resilience to stress (Arnold et al., 2019). When females mate in traditional mating chambers, an increase in anxiety-related behaviors is observed, without behavioral changes in the PM group (Nyuyki et al., 2011). In the study by Nyuyki and colleagues, anxiety-related behaviors were measured in the elevated plus maze and the black-white box tests after a 30-min mating test. Hormonal priming induced anxiolytic effects, compared to non primed females, when the females mated in PM. On the other hand, mating under NP abolished this anxiolytic effect of hormonal priming. Additionally, primed rats that underwent NP showed higher anxiety-related behaviors than primed rats that experienced PM. The same authors also showed that oxytocin (OT) is released in the paraventricular nucleus of the hypothalamus in the PM group but not in the NP condition. The administration of an OT antagonist partially prevented the anxiolytic effects of PM. These results indicate that PM triggers activation in the OT system that enhances anxiolysis by sex steroids that might facilitate the establishment of sexual reward, reducing the aversive components of mating through this protective-stress effect.

### 6.3. Dopamine

Dopamine (DA) is another neurotransmitter released during PM conditions, as evaluated by *in vivo* microdialysis. The authors placed a cannula in the *nucleus accumbens* (NAc) and monitored minute-by-minute DA levels during mating tests. They only found a peak of extracellular DA before the first intromission in the PM group. The authors proposed that DA is released in response to cues associated with predicting a rewarding state induced by PM compared with NP (Jenkins and Becker, 2003b). However, there is evidence that DA does not participate in the rewarding properties of sexual behavior. For example, DA antagonists do not block the reward state induced by sexual behavior in males (Agmo and Berenfeld, 1990) or females (Garcia Horsman and Paredes, 2004). In fact, it has been suggested that DA induces generalized behavioral arousal (Alcaro et al., 2007). Moreover, a series of studies evaluating the role of DA in reward by Berridge and Robinson have shown the DA does not mediate hedonic pleasure of reinforces, “DA systems appear necessary for wanting incentives but not for liking them” (Berridge and Robinson, 1998). Whatever the neuromodulator involved, it is clear, as demonstrated by several groups, that sexual behavior induces a reward state. Although opioids are the most likely candidates, DA and OT could also participate directly or indirectly through an interaction with the opioid system in the reward state induced by sexual behavior in males and females observed in rats (Miller and Baum, 1987; Agmo and Berenfeld, 1990; Hughes et al., 1990; Mehrara and Baum, 1990; Oldenburger et al., 1992; Paredes and Martinez, 2001; Kippin and van der Kooy, 2003; Harding and McGinnis, 2004; Meerts and Clark, 2007; Paredes, 2014), mice (Kudwa et al., 2005) and hamsters (Meisel and Joppa, 1994; Bell et al., 2010).

### 6.4. Gene expression

In addition to the endocrine responses, mating also modifies gene expression differentially according to mating conditions. A report evaluating the immediate early gene expression by FOS immunoreactivity (FOS-IR) 1 h after receiving 5 or 15 intromissions under NP and PM in rats found increased FOS-IR in brain areas relevant for reproduction, such as the MPOA, the VMH, and the bed nucleus of the stria terminalis (BNST) in both NP and PM groups compared with controls and females which received mounts only and those who stay in their home cage. In contrast, the posterodorsal medial nucleus of the amygdala (MePD) showed increased FOS-IR only in the PM group proportionally with the number of intromissions. The authors proposed that the MePD is a region that receives inputs from other areas and serves as a center to modulate behavioral and neuroendocrine responses induced by mating (Erskine and Hanrahan, 1997).

## 7. Behavioral pharmacology of paced mating

As mentioned, PM mating is a feasible way to evaluate neurobiological mechanisms of sexual motivation and reward in females. For this reason, its use is extended in preclinical trials to explore its validity in human conditions, mainly regarding sexual dysfunctions. In the following section, we will describe how paced mating has been used to evaluate different compounds and doses in combination with other methods as a valuable tool to study different aspects of sexual behavior and motivation. The growing interest in new pharmacological agents for treating female sexual dysfunctions, mainly associated with the motivational components, makes the PM method ideal for dissecting drug effects, especially those affecting mood and anxiety.

### 7.1. Psychotropic drugs and PM

A study evaluated sexual and anxiety-like behaviors after weekly administration (4 in total) of ketamine (10 mg/kg/i.p.) in PM conditions. Females in the ketamine group spent more time in the male’s compartment. They showed a reduced percentage of exits after a mount and shorter latencies to return to the male side after an intromission compared with controls. However, the effects were attenuated by sexual experience (Guarraci et al., 2020). In addition, ketamine did not affect anxiety-like behavior in the elevated plus maze test. The authors proposed that ketamine elevated the pain threshold in females. This effect could diminish the aversive components during mating. They also evaluated if previous sexual experience could influence the effect of a single dose of fluoxetine or ketamine in female rats in PM. The fluoxetine group spent less time in the male’s compartment and showed longer return latencies after ejaculations, whereas ketamine did not modify sexual behavior (Marshall et al., 2020).

Serotonin elicits bimodal effects on sexual behavior in females, depending upon the receptor subtype involved. For example, 5-HT_1A_ and 5-Ht_1B_ agonists inhibit proceptivity and receptivity in Ovx hormonally primed female rats, whereas 5-HT_2_ and 5-HT_3_ stimulation facilitate lordosis behavior and its antagonism provokes the opposite (Snoeren, 2019). However, most studies evaluating the role of different neurotransmitters in female sexual behavior have been done in NP conditions. As aforementioned, the mating condition is crucial since the effects could be other if the female controls or not the sexual interaction. For example, a study on the effects of the chronic treatment of paroxetine (10 or 20 mg/kg/p.o. 56 days) in Ovx hormonally sub-primed and fully primed females tested under PM showed no changes in any sexual behavior parameters for four 30-min sexual behavior tests (once a week). After day 21 of treatment, females also received weekly doses of the 5-HT_1A_/5-HT_7_ receptor agonist 8-Hydroxy-2-(dipropylamino) tetralin hydrobromide (8-OH-DPAT, 0.1 or 0.3 mg/kg/i.p.), alone and in combination with the selective 5-HT_1A_ antagonist WAY-100635 (0.3 mg/kg/s.c.). Sexual behavior was tested 30 min after those treatments to evaluate the possible 5-HT_1A_ desensitization by chronic paroxetine. The 8-OH-DPAT agonist reduced, in a dose dependent manner, proceptive behaviors in sub-primed and fully primed groups treated with vehicle and showed a right-shift dose–response curve in females treated with paroxetine, indicating receptor desensitization, whereas cotreatment with WAY100635 counteracted these inhibitory effects. The results indicate that chronic paroxetine treatment does not modify sexual behavior in females in PM, even after 5-HT1A desensitization (Snoeren et al., 2011).

### 7.2. Psychomotor active drugs and PM

The repeated administration of some psychoactive drugs facilitates the rewarding effects of mating (indicative of cross-sensitization). Studies showed conflicting results in female rats in PM tests. For example, a single dose of d-amphetamine (1.0 mg/kg, i.p.) increased the percentage of exits following mounts and intromissions. When rats received *d*-amphetamine chronically (1.0 mg/kg, i.p. daily for 3 weeks) and were tested 1 week after the final injection, they showed shorter latencies to return after mounts than controls (Afonso et al., 2009). Another report showed that females displayed more proceptive behaviors in a bilevel chamber test 21 days after the last of three doses of *d*-amphetamine (1 mg/kg, i.p. every other day). Results suggest that there is a cross-sensitization by *d*-amphetamine and sexual behavior in females after a chronic treatment that is not explained by increased locomotor activity since those effects are presented after a washout period. However, when *d*-amphetamine is infused in the NAc (40 μg/0.5 μL), there is a lack of effect in PM. In contrast, infusion into the MPOA (10 μg/0.5 μL) showed similar effects to those presented after an acute administration. After an MPOA lesion, i.e., females spend less time in the male’s compartment and leave the male side more frequently after receiving a mount without affecting lordosis (Guarraci and Bolton, 2014). The authors proposed that d-amphetamine infused directly into the MPOA causes excessive dopaminergic activity and the inhibition of sexual behavior.

The effects of Methamphetamine (MA) on PM have also been evaluated since its use increases sexual activities, including those associated with high risks, especially in women. Ovariectomized and hormonally primed rats received three doses of MA (5 mg/kg/i.p./day). Four hours after the last MA injection, rats were tested in PM tests that lasted 25 min to avoid locomotor effects. Females in the MA group showed shorter MRL, ERL, and reduced %E after intromissions. Females also displayed more proceptive behaviors and reduced rejection components. Moreover, their LQ and LI were higher compared with controls. Additionally, MA increased spinophilin protein expression (a dendritic spine density marker) in the medial AMG, suggesting neuroplastic changes in synaptic transmission. However, it is unclear if the plastic change is induced by sexual behavior itself because NP was not evaluated (Holder and Mong, 2010). To assess the possible cross-sensitization by MA, they evaluated PM in female rats 21 or 6 days after the last injection in two schemes of chronic MA (1 mg/kg/every other day for a total of 3 days, or 1 mg/kg/daily/for 12 consecutive days). Methamphetamine did not modify sexual behavior when tested after 6 or 21 days of abstinence. Results indicate that MA failed to induce a cross-sensitization with sexual behavior (Thibodeau et al., 2013).

Methamphetamine might enhance sexual motivation in females depending on its interaction with the excitatory dopamine receptor subtype 1 (DR1) and progestins in the MePD, a brain site where multisensory sexually-relevant stimuli and generalized arousal increase the incentive value for a sexual partner (Rudzinskas et al., 2019). It is proposed that MA induces DA release in the MePD that activates DR1. This activation favors the estrogen receptors (ER) translocation to the nucleus, increasing the progesterone receptors (PR) transcription in a ligand-independent manner. In this way, MA increases P sensitivity by up-regulating PR to facilitate sexual motivation even in subthreshold doses of P (Rudzinskas et al., 2019). The facilitatory role of P and its metabolites in establishing sexual reward induced by PM in females was previously reported after their i.v. administration (Gonzalez-Flores et al., 2004).

Another psychomotor active drug tested in PM is caffeine. A single moderate dose of this substance (15 mg/kg/s.c.) reduced the return latency after ejaculation and increased motor activity. In a partner preference test, females in the caffeine group also visited more times the male than the female, although the time spent in the male’s compartment was similar to the control group. The results indicate that caffeine induces a general activation that might secondarily stimulate approach behavior to a sexual partner (Guarraci and Benson, 2005).

### 7.3. Prosexual drugs and PM

Paced mating has been used to evaluate drugs with potential effects on sexual activity in preclinical studies. For example, the phosphodiesterase type-5 (PDE-5) inhibitor, zaprinast, which increases genital blood flow in women, was tested in rats (Clark et al., 2009). They received one of three different doses (1.5, 3, and 6 mg/kg/i.p.) 20 min before testing. Zaprinast increased contact-return latency after ejaculation in a dose–response fashion without modifying receptivity. The authors proposed that since zaprinast enhances blood supply, it also increases vaginal sensitivity, which explains the increase in contact return latencies.

Another drug tested is PT-141, a melanocortin receptor agonist, to evaluate its effects on female sexual behavior. Different groups of females received doses of 50, 100, and 200 μg/kg/ml/s.c. of PT-140 5 min before PM tests (30 min duration) in unilevel or believer chambers. Females in the 100 and 200 μg/kg groups showed increased proceptive behaviors in both types of tests without affecting lordosis or pacing measurements. The authors concluded that PT-141 enhances solicitation in females, which indicates that the melanocortin central system is relevant for sexual motivation (Pfaus et al., 2004).

Unfortunately, no pharmacological studies have directly compared a particular drug’s effects in both PM and NPM. This is important for future studies in which the pharmacological effects of a drug want to be evaluated on sexual behavior, considering the physiological and behavioral differences induced by PM and NPM.

## 8. PM under different animal models of human diseases

Some studies have used PM as a model to study sexual dysfunctions in women since it is possible to dissociate motivational and consummatory aspects after pharmacological treatments.

### 8.1. Pacing and nociceptive conditions

Vulvar pain is an underdiagnosed condition that affects around 10% of women and disrupts sexual function, but also causes high levels of distress and interpersonal difficulties (Farmer et al., 2014; Schlaeger et al., 2019; Chisari et al., 2021; Torres-Cueco and Nohales-Alfonso, 2021). For these reasons, different methods, including PM, have been used to study its physiopathological mechanisms. Farmer et al. (2014) evaluated if pain reduces sexual motivation in female mice using an arena divided into two compartments with four holes at the bottom to pace the sexual interaction. They used a model of inflammatory pain induced by injecting (1) zymosan A (0.5 mg/mL/10 μL, s.c.) into the genital area (center-posterior vulva or center-dorsal penile shaft) or in the hind paw, or (2) 2% λ-carrageenan (s.c. dissolved in 10 μL of saline) into the right cheek or the ventral tail. In that way, they evaluated different combinations of inflammatory pain inducers and areas. Four hours after injecting one of the compounds and confirming the induction of the nociceptive response, female sexual behavior was observed for 1 h under PM conditions. Females received fewer mounts in all groups with inflammatory pain, spent less time in the male’s compartment, and displayed fewer proceptive behaviors, compared with pain-free controls, without affecting receptivity. These effects were reversed with the treatment of an anti-inflammatory (pregabalin) or a prosexual drug (apomorphine or melanotan II; Farmer et al., 2014). On the other hand, male sexual behavior was unaffected in all pain induced groups. These results indicate that there are sex differences in the incentive value of mating towards an aversive context, i.e., pain. In females, sexual incentive motivation is inhibited, whereas, in males, pain is overcome by mating.

In another report regarding pain-related conditions and PM behavior, females were implanted with an autologous endometriotic tissue in the intestine of two experimental groups of female rats. One group was Ovx and hormonally primed. The second group was maintained on natural proestrus, and both were tested 50 days after the implant under PM conditions. There were no differences in the percentage of exits or in the return latencies after mounts, intromissions, or ejaculations. However, the amount of endometriotic tissue implanted was positively correlated with the contact return latency following ejaculation. The authors concluded that endometriotic implants slightly modify the sensitivity to vigorous sexual stimulation (Clark et al., 2011). However, that study did not corroborate the presence of vaginal hypersensitivity to confirm that subjects felt pain.

### 8.2. Pacing and hyperglycemic conditions

The administration of streptozotocin (STZ), a toxin that induces the death of beta cells in the pancreas, depletes insulin production, that causes hyperglycemia. When STZ is administered neonatally, insulin depletion is partial, with mild glucose elevation in the blood. If STZ is administered in adult rats, the deficit is even higher, inducing severe hyperglycemia. A study evaluated sexual behavior in rats using both STZ models under PM and NP conditions. The results showed a reduction of LQ in females with severe hyperglycemia tested in NP conditions, whereas, in the mild hyperglycemia group, there were no changes in the LQ or LI in NP and PM groups (Hernandez-Munive et al., 2019). However, pacing behavior was disrupted in the PM group since females did not exit the male compartment after receiving a mating stimulus. A possible explanation for this result is a sensory disruption induced by the persistent hyperglycemic state that leads to decreased neuronal activity in the lumbosacral dorsal horn in female rats treated with streptozotocin (Nakagawa et al., 2020). The number of aggressions (boxing, bites, and lateral postures) was higher in the NP condition, than in the PM condition. The authors proposed that the aversiveness of the NP condition favored rejection behaviors towards the male. The exogenous supplementation of insulin prevented aggressive behavior in females. The results suggest that the hyperglycemic state induces changes in vascularity and morphology of the vaginal tissue (Kim et al., 2006), neuropathy (Tripathi et al., 2016) and increases anxiety-like behaviors (Aksu et al., 2012). These factors together disrupt the execution of PM, increasing the aversive components of mating (Hernandez-Munive et al., 2021).

Most of the early studies done to evaluate the effects of different neurotransmitters or pharmacological compounds upon sexual behavior were done in traditional mating chambers where the male controls the sexual interaction. From the above described studies is clear that different compounds and doses modify sexual behavior depending if they were tested under PM or NP conditions. By using PM, the authors can reduce the aversive components of mating and increase the appetitive aspects of this behavior, dissociating the motivational aspects of mating. These conditions resemble what occurs in natural and seminatural conditions, and it is a better model to approach the study of sexual dysfunctions (Table 1).

**Table 1:** Differences between paced mating (PM) and non-paced mating (NP) in different parameters of female rats.

Parameter	Comparison	Interpretation	References
Litter size	More pups in PM than NP	PM favors reproduction	Coopersmith and Erskine (1994)
Prolactin surges induced by mating	Higher in PM than NP	PM induces prolactin release favoring reproduction	Erskine and Kornberg (1992)
Rejection behaviors	Higher in NP than in PM	Paced mating is less aversive	Erskine (1989)
CPP: reward state induced by PM	Consistently induced by PM under different conditions	PM facilitates sexual reward even when mating is extended	Paredes and Alonso (1997) and Arzate et al. (2011)
CPP: reward state induced by NP	Weak effect CPP only when males ejaculated after few intromissions	CPP not consistently induced Males ejaculating after few intromissions have not been observed by other groups	Oldenburger et al. (1992) and Meerts and Clark (2007)
Anxiety	PM does not increase basal anxiety, while NP increases anxiety-related behaviors PM reduces post-stress anxiety Steroid-primed rats exposed to PM show lower anxiety than steroid-primed rats exposed to NP	NP is anxiogenic, while PM is not PM confers resilience to stress PM, but not NP, allows progesterone-mediated anxiolysis	Arnold et al. (2019) and Nyuyki et al. (2011)
FOS immunoreactivity	Increase FOS-IR in the MPOA, the VMH and the BNST in NP and PM. Increased FOS-IR in the MePD only in PM	MePD modulates qualitative as well as quantitative aspects of PM	Erskine and Hanrahan (1997)
Neurogenesis Rostral Migratory stream Accessory olfactory bulbs Main olfactory bulbs	PM and NP induce more cells More neurogenesis 15 and 45 days after PM More neurogenesis 15 and 45 days after repeated PM	PM induces long-term neuroplastic changes mediated by the endogenous opioid system. PM and NP induce long-term neuroplastic changes when the stimulation is repeated.	Alvarado-Martínez and Paredes (2018), Bedos et al. (2018), and Portillo et al. (2020)

CPP, conditioned place preference; MPOA, medial preoptic area; VMH, ventromedial hypothalamus; BNST, bed nucleus of the stria terminalis; MePD, posterodorsal medial amygdala.

## 9. Magnetic resonance imaging studies and PM

In recent years, imaging studies have been growing since this technique is non-invasive and can be used in longitudinal protocols to evaluate anatomical and functional changes over time. Manganese-enhanced magnetic resonance imaging (MEMRI) enables the evaluation of neural activity since manganese is an analog of calcium which enters and accumulates in neurons with their depolarization. Additionally, since manganese’s paramagnetic properties enhance contrast in T1 weighted images, its accumulation is related to neuronal activation. Using MEMRI, our group evaluated the activity of several brain areas immediately after 1, 5, and 10 PM sessions (one test per week) in Ovx and hormonally primed female rats. The results showed no differences after the first PM test in all the groups. After the 5th PM test, the signal intensity increased in areas related to the sociosexual behavior circuit, such as the OB, the bed nucleus of the stria terminalis (BNST), the AMG, the MPOA, and the VMH. This increase continued at session 10. On the other hand, areas associated with the reward circuit (the NAc, the striatum, the hippocampus, and the VTA) showed no changes until session 10th. This was the first longitudinal study demonstrating that the sociosexual circuit is activated before and with a higher intensity than the reward circuit, and this activation is modified by sexual experience (Aguilar-Moreno et al., 2022). Further studies will evaluate if the motivational and consummatory aspects of sexual behavior activate different brain circuits.

## 10. PM and animal welfare

Increasing attention is being devoted by researchers to the issue of animal welfare among laboratory rodents and of refinement of behavioral procedures (Jirkof et al., 2019; Voikar and Gaburro, 2020; O'Malley et al., 2022; d’Isa and Gerlai, 2023). From the evidence described above it is clear that PM improves animal welfare compared to NP. As already described, PM favors reproduction by inducing a higher release of luteinizing hormone and prolactin (Erskine and Kornberg, 1992), increases the probability of the females getting pregnant, and those pregnant females sire more pups than females in the NP condition (Erskine, 1985, 1989). Additionally, while NP increases anxiety-related behaviors, PM does not (Nyuyki et al., 2011). Importantly, PM reduces anxiety after an acute stressor, suggesting that PM confers resilience to stress (Arnold et al., 2019). Moreover, PM induces a reduction in rejection and aggressive behaviors compared to NP (Erskine, 1989). Furthermore, females can pace the sexual interaction in a 1 h test or longer without an increase in aggressive and rejection behaviors, as is usually observed in NP tests (Arzate et al., 2013; Ventura-Aquino and Fernández-Guasti, 2013). Another essential advantage of PM is that, under this condition, sexual behavior is rewarding in male and female rodents (Martinez and Paredes, 2001). This reward state is mediated by opioids in males and females (Paredes, 2014). Together, these findings indicate that PM improves animal welfare compared to NP, representing a valuable refinement of rodent sexual behavior models.

## 11. Conclusion

The development of the friendly PM methodology was crucial to improve our understanding of the physiological and behavioral consequences associated with the possibility of controlling the rate of sexual stimulation. It also demonstrated that females could discriminate the intensity of the stimulation they received, either a mount and intromission or an ejaculation. It has also contributed to the study of the motivational and rewarding properties of mating in females. It can also be used to study long term plastic changes, neurogenesis induced by PM. The interest in PM is growing since the complexity of its elements and consequences at different levels makes this paradigm a valuable tool for studying physiological, behavioral, motivational, rewarding, and plastic changes in a laboratory setting resembling natural conditions. Moreover, PM is also employed to evaluate new pharmacological strategies to treat female sexual dysfunctions that could impact sexual health. However, we must cautiously extrapolate the results obtained in PM studies to the human clinical field.

## Author contributions

All authors listed have made a substantial, direct, and intellectual contribution to the work, and approved it for publication.

## Funding

This research was supported by PAPIIT UNAM grant IN206521.

## Conflict of interest

The authors declare that the research was conducted in the absence of any commercial or financial relationships that could be construed as a potential conflict of interest.

## Publisher’s note

All claims expressed in this article are solely those of the authors and do not necessarily represent those of their affiliated organizations, or those of the publisher, the editors and the reviewers. Any product that may be evaluated in this article, or claim that may be made by its manufacturer, is not guaranteed or endorsed by the publisher.
