# Erratum: Being friendly: paced mating for the study of physiological, behavioral, and neuroplastic changes induced by sexual behavior in females

**DOI:** 10.3389/fnbeh.2023.1327758

**Published:** 2023-11-07

**Authors:** Frontiers Production Office

**Affiliations:** Frontiers Media SA, Lausanne, Switzerland

**Keywords:** paced mating, positive affective state (reward), motivation, opioids, rats

Due to an error, the caption of Figure 1 contained two typos. It was originally published as:

“For experimental strategies use to evaluate paced mating in female rats.”

“For” has been corrected to “Four” and “use” to “used”. The correct sentence appears below.

“Four experimental strategies used to evaluate paced mating in female rats.”

The publisher apologizes for this mistake.

The original article has been updated.

